# Optimal strategies for throwing accurately

**DOI:** 10.1098/rsos.170136

**Published:** 2017-04-26

**Authors:** M. Venkadesan, L. Mahadevan

**Affiliations:** 1 Department of Mechanical Engineering and Materials Science, Yale University, New Haven, CT 06520, USA; 2 Department of Physics, Paulson School of Engineering and Applied Sciences, Harvard University, Cambridge, MA 02138, USA; 3 Department of Organismic and Evolutionary Biology, Paulson School of Engineering and Applied Sciences, Harvard University, Cambridge, MA 02138, USA

**Keywords:** throwing, noise propagation, optimal planning, speed–accuracy trade-off

## Abstract

The accuracy of throwing in games and sports is governed by how errors in planning and initial conditions are propagated by the dynamics of the projectile. In the simplest setting, the projectile path is typically described by a deterministic parabolic trajectory which has the potential to amplify noisy launch conditions. By analysing how parabolic trajectories propagate errors, we show how to devise optimal strategies for a throwing task demanding accuracy. Our calculations explain observed speed–accuracy trade-offs, preferred throwing style of overarm versus underarm, and strategies for games such as dart throwing, despite having left out most biological complexities. As our criteria for optimal performance depend on the target location, shape and the level of uncertainty in planning, they also naturally suggest an iterative scheme to learn throwing strategies by trial and error.

## 1. Introduction

Accurate throwing is a skilled motor task in humans that has inspired many studies of motor control [1–6]. However, robust and commonplace observations such as the trade-off between speed and accuracy [5,7–11], or the choice of overarm versus underarm styles depending on the target, remain unexplained. It is possible that these features are consequences of the underlying biological complexity associated with planning and execution. For example, the intensity of noise in muscles depends on their force output [6,11,12] thereby leading to a speed–accuracy trade-off, and the choice of throwing style may simply be idiosyncratic.

However, a complementary perspective on the problem is that the physical dynamics of projectile flight map the variability in initial conditions to that of the landing location. This approach to error propagation and amplification in dynamical systems has antecedents that go back more than a century [13], but continue to have implications for prediction and decision-making. The specific example of throwing is particularly interesting because it decouples the internal (neural/cognitive) decision from the dynamics of a projectile, separating planning from control. Once the projectile has been launched, there is no possibility of control, differentiating this task from much better studied tasks such as pointing and tracking [14]. Instead, one has to learn strategies from an iterative process of error estimation and correction from one trial to the next. This has led to studies of error propagation via the throwing actions used in specific sports, such as basketball [4,15,16], darts [3] or *pétanque* [1], where the analyses treat throwing as a problem of shooting, i.e. the arm plays no role. Here, we complement these by studying optimal strategies in throwing using a simple model of the arm as a finite object, and an analysis of error propagation through the dynamics of an ideal projectile flight. This allows us to address the qualitative selection of overarm versus underarm styles, as well as the quantitative selection of the release angle and speed, and their dependence on the target geometry, location and throwing speed. We then use our results to analyse some experiments in the context of games that involve throwing, and also consider the role of structured noise in the release parameters to determine how this plays out in determining optimal strategies. We finally look at the role of planning uncertainty in characterizing how errors are amplified, and what this implies for a measured approach to learning the optimal strategy for throwing.

## 2. Mathematical model

In a minimal setting, we model the arm as a rigid hinged bar that releases a drag-free point projectile at any desired angle *ϕ* and angular speed *ω*. We note that the choice of an angle and angular velocity as the primary variables is deliberate; prescribing an alternate set of variables on a cartesian system are not completely equivalent. This is because the choice of basic variables play an important role in determining how errors are amplified via the Jacobian of the transformation from one basis set to another. Our choice is ego-centric, centred about the thrower using a natural angular set of variables; other choices and how they affect error propagation will yield different quantitative results. However, we show how behaviours like the speed–accuracy trade-off are robust to the choice of release parameters.

The variability and correlations in the release parameters (*ϕ*,*Ω*) depend on the detailed properties of the neuromuscular system. Here, we assume that the noise in *ϕ* and *Ω* are uncorrelated; in the context of a linearized analysis, we need no further assumptions about the specific distributions associated with the noise. We assume that the goal of the arm is to throw the projectile into a bin, modelled as a target that presents an up-facing horizontal surface with its centre at a distance ℓ and height *h* away from the base of the arm. Lengths are expressed in units of the arm length and accelerations in units of Earth’s gravitational acceleration. The dynamics for the position of the projectile *x*(*t*),*y*(*t*) are:

2.1 $$\begin{array}{rl} \ddot{x} & =0,\;\ddot{y}=-1,\\ x(0) & =\cos\phi ,\;\dot{x\;}\;(0)=\omega \sin\phi \\ \;\text{and}\;y(0) & =\sin\phi ,\;\dot{y\;}\;(0)=-\omega \cos\phi . \end{array}\}$$

There is a one parameter family of throwing strategies, found by solving for three unknowns (*t*,*ϕ*,*ω*) using the two equations (2.1), which satisfy the relations for the projectile to exactly strike the target at ℓ,*h*:

2.2*a* x(t,ϕ,ω)=cos⁡ϕ−tωsin⁡ϕ=ℓ

and

2.2*b* $$y(t,\phi ,\omega )=\sin\phi +t\omega \cos\phi -\frac{1}{2}t^{2}=h.$$

To enter the bin, the projectile must move downwards at the time *t*_*h*_ corresponding to the projectile reaching the height *h*, i.e. $\dot{y}(t_{h})=\omega \cos\phi -t_{h}\leq 0$. Using this condition, and solving (2.2b), the time of impact is given by $t_{h}(\phi ,\omega )=\omega \cos\phi +\sqrt{\omega^{2}\cos^{2}\phi -2(h-\sin\phi )}$. The horizontal position *x*_*h*_ of the projectile as it strikes the plane of the target is then given by

2.3 $$x_{h}(\phi ,\omega )=\cos\phi -\omega \sin\phi \left(\omega \cos\phi +\sqrt{\omega^{2}\cos^{2}\phi -2(h-\sin\phi )}\right).$$

**Figure 1.**
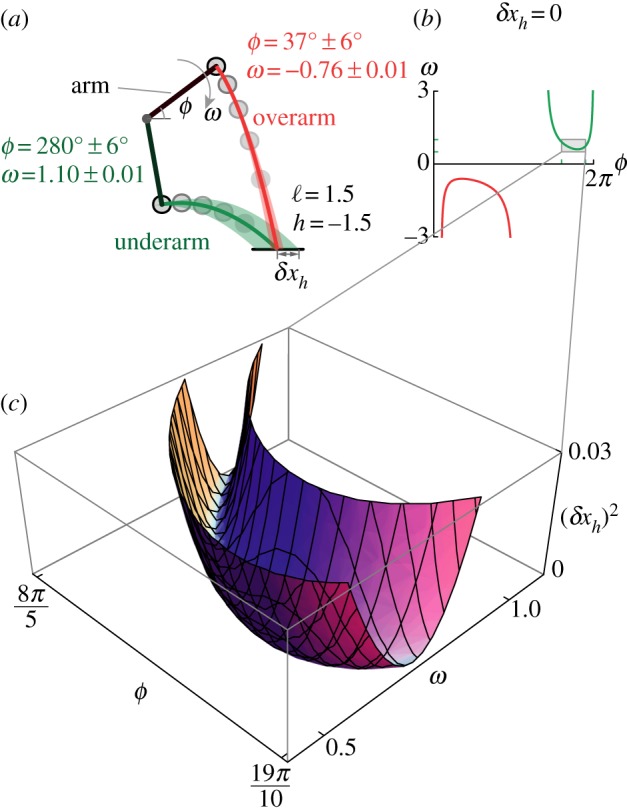
Error propagation in throwing depends on the trajectory of the projectile. Underarm=*ω*_0_(*ϕ*)>0; overarm=*ω*_0_(*ϕ*)<0. (*a*) For a given target, there are generally multiple ways to strike it exactly; the solid red and green trajectories are two examples using an overarm and underarm style, respectively. The lightly shaded bands shows how uniformly distributed errors in position and velocity propagate; the overarm throw is more accurate. (*b*) The curve of launch parameters (*ϕ*,*ω*_0_(*ϕ*)) given by equation (2.4) that exactly strike the target. (*c*) Deviations away from this curve leads to an error (*δx*_*h*_)^2^ as a function of *ϕ* and *ω*. Error amplification is quantified by the maximal curvature of the valley of this surface.

In figure 1*a*, we show two such choices corresponding to the solid red and green trajectories for throws into a horizontal bin with its centre located at ℓ=1.5, *h*=−1.5, i.e. in front of and below the shoulder. Naturally, there are some target positions and arm postures which are disallowed, and characterized by the requirements that $\omega^{2}\geq 2(h-\sin\phi )/\cos^{2}\phi$ and h≥sin⁡ϕ (see the electronic supplementary material for details). Setting *x*_*h*_(*ϕ*,*ω*)=ℓ, we obtain the one-parameter continuum of strategies for exactly striking the centre of the target, as given by the speed–angle relationship,

2.4 $$\omega_{0}(\phi )=\frac{\cos\phi -\ell }{\sin\phi \sqrt{\frac{2}{\sin\phi }(1-\ell \cos\phi -h\sin\phi )}},$$

Figure 1*b* shows the solution curve *ω*_0_(*ϕ*) for the target shown in figure 1*a*. In general, there will be errors in the final position, which we define to be *δx*_*h*_=*x*_*h*_−ℓ, the difference in the distance traversed *x*_*h*_ and the target distance ℓ when the projectile reaches the height *h*. To choose from this continuum of possible trajectories, we need a criterion. Noting that there will always be errors in the initial condition, we suggest that the best strategy from this one parameter family of solutions is one that is maximally tolerant of these initial errors.

## 3. Min-max strategy for optimal throwing

To quantify the amplification of small launch errors, we linearize *x*_*h*_(*ϕ*,*ω*) in the neighbourhood of the curve (*ϕ*,*ω*_0_(*ϕ*)) to obtain the relationship between the ‘input error vector’ *ϵ*=(*δϕ* *δω*) and the ‘output/target error’ *δx*_*h*_ given by *δx*_*h*_≈*J*_err_(*ϕ*)⋅*ϵ*, where

3.1 $$J_{\mathrm{err}}(\phi )={\left(\begin{array}{cc} \frac{\partial x_{h}}{\partial \phi } & \frac{\partial x_{h}}{\partial \omega } \end{array})\right|}_{\phi ,\omega_{0}(\phi )}.$$

In fact, as there is a one-dimensional curve of solutions (*ϕ*,*ω*_0_(*ϕ*)), where *δx*_*h*_=0, *J*_err_ will be rank deficient, i.e. it has a zero singular value and an associated non-trivial null-space, namely, the tangent to the curve (*ϕ*,*ω*_0_(*ϕ*)), while the other singular value is λ(*ϕ*). In figure 1*a*, we show that the amplification of errors in the release angle *δϕ* and speed *δω* exemplified by the lightly shaded bands depends upon the trajectory of the projectile; the overarm throw is clearly the better choice here. In figure 1*c*, we show how the squared-error $\delta x_{h}^{2}(\phi ,\omega )$ varies as a function of the uncertainty in the release parameters. The minimum (valley) is simply the solution curve *ω*_0_(*ϕ*), and the maximum curvature of the surface orthogonal to this valley is a measure of how $\delta x_{h}^{2}$ grows for small launch errors *δϕ* and *δω*. It is easy to see that the curvature of the error surface is 2λ^2^, where λ is the non-zero singular value of the Jacobian that maps the initial conditions to the final state. Errors in *ϕ* and *ω* that are tangent to the solution curve *ω*_0_(*ϕ*) cancel each other by virtue of belonging to the null space of the error propagation map *J*_err_, otherwise they are amplified in proportion to λ. Thus, the reciprocal of λ is a natural measure of accuracy, henceforth denoted by *p*, i.e. *p*=1/λ.

### 3.1. Accurate throwing

**Figure 2.**
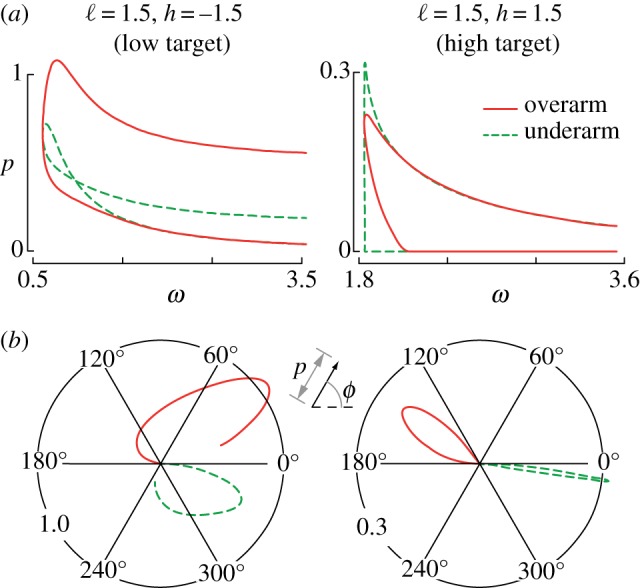
Error amplification for throwing depends on the target location and planning errors. (*a*) For two different targets, one below and another above the shoulder, a comparison between all possible overarm (solid red) and underarm (dashed green) throws. Accuracy is quantified by the inverse of the non-zero singular value, i.e. *p*=1/λ of the map *J*_err_ given by equation (3.1) as a function of the release velocity *ω*. (*b*) Polar plots of accuracy *p*(*ϕ*)=1/λ(*ϕ*) as a function of arm angle at release *ϕ* for the same two different targets. We see that even if *p*(,*ω*_*m*_,*ϕ*_*m*_) is a maximum, it can fall off quickly, so that such a strategy is susceptible to small planning errors, i.e. inaccuracies in the internal model of the dynamics.

Accuracy *p* is parametrized either using the launch speed *ω* by considering the neighbourhood of the function $\omega_{0}^{-1}(\omega )$ (inverse of (2.4)), or using the launch angle *ϕ* by considering the neighbourhood of the solution curve *ω*_0_(*ϕ*). In figure 2*a*, we show four curves that quantify accuracy for two given targets; for each, there are two possible overarm throws (shallow or high) and similarly for underarm throws. For each of the example targets, faster throws lead to a sharp decay in accuracy, and the overarm throw is as good or better than the underarm throw at high speeds. In figure 2*b*, we show polar plots of the accuracy (radial distance) as a function of launch angle (polar angle). For the target below the shoulder with *h*=−1.5, the overarm throw is more accurate than underarm. The converse is true for the second example of a target above the shoulder with *h*=1.5. However, for the second target, the superiority of the underarm throw is extremely sensitive to any uncertainty in planning because of its very sharply peaked shape. Planning uncertainties would manifest as an incorrect choice of optimal release parameters, and the underarm strategy would be sensitive to these planning errors.

## 4. Speed–accuracy trade-off

Speed–accuracy trade-offs in biological systems are usually explained as the result of structured noise in the system dynamics [11,17] in the form of corrective submovements, velocity-dependent noise, activation-dependent noise or more generally ‘signal-dependent’ and structured covariance of input noise at the level of muscles [10,12,18]. Given that our physical picture for throwing introduces noise in a simple setting, we ask what the consequences are for speed–accuracy trade-offs.

**Figure 3.**
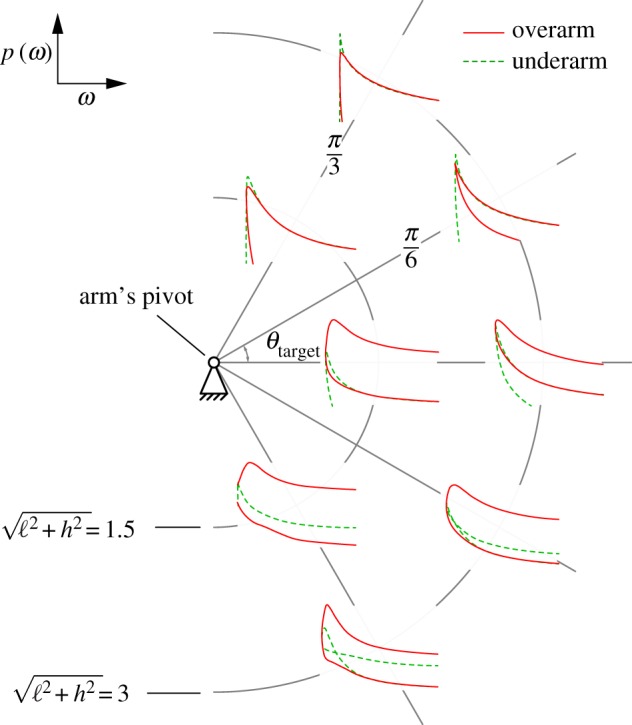
Speed–accuracy trade-off for various locations of a horizontal target. The location of each set of curves corresponds to the target location. The target locations are specified by the angle to the target *θ*_target_ and distance $\sqrt{l^{2}+h^{2}}$. For each target location and choice of throwing speed (absolute value), there are generally four distinct release angles: either overarm or underarm, and for each style, either a shallow (closest to straight-line path) or high throw. Every curve shows a decrease in accuracy for higher speeds. For most target locations, our model predicts that the most accurate throw is at speeds slightly higher than $\omega_{\min}$ the smallest possible speed to reach the target. Of all curves at each target location, a shallow overarm throw is typically more accurate than the other three styles at all speeds. All calculations restricted $\omega_{0}\in [\omega_{\min},3.54]$; for a 1 m arm length, this corresponds to a maximum launch speed of ≈11.1 m s^−1^≈40 km h^−1^.

In figure 3, we show that slower throws are typically more accurate than faster ones—the classic trade-off between speed and accuracy that is observed in multiple contexts of human motor behaviour [7–10,12,18,19]. The most accurate throw is typically slightly faster than that associated with the minimum speed $\omega_{\min}(\ell ,h)$ needed to reach a given target. At higher speeds, the shallow overarm throw is most accurate, particularly for targets at or below the arm pivot (equivalent to the shoulder). Therefore, the physics of projectile flight dictates that throwing slowly generally maximizes accuracy, and if it becomes necessary to throw fast, an overarm style is the more accurate one. The speed–accuracy trade-off is also seen for other target geometries such as a vertically oriented target, as shown in the electronic supplementary material.

### 4.1. Generalized speed–accuracy trade-off

**Figure 4.**
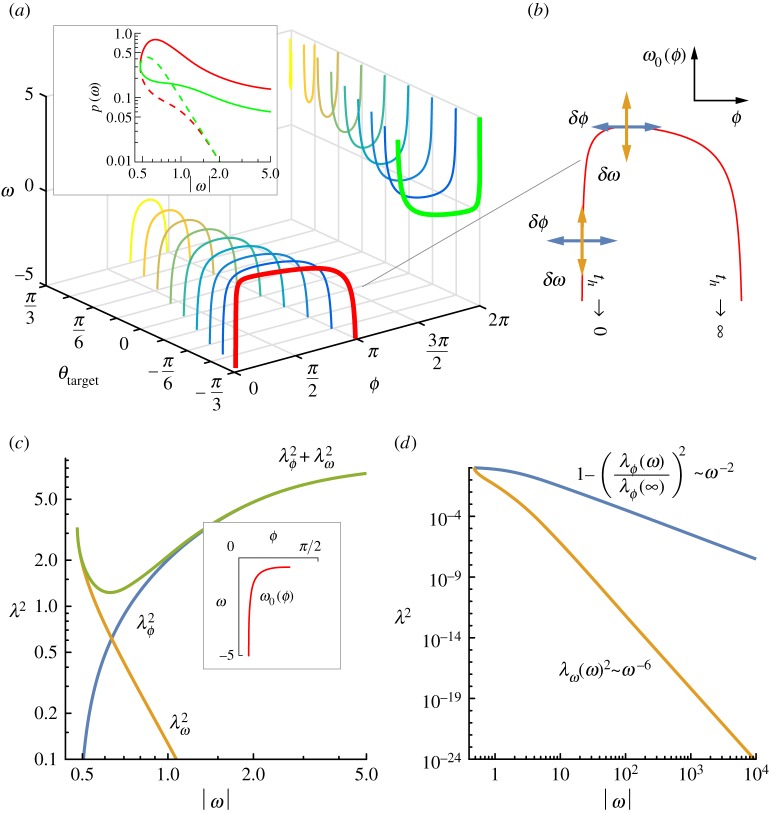
The speed–accuracy trade-off emerges independent of the target location, and the relative noise level in release angle versus speed. (*a*) Speed versus angle plots for targets at various positions around the arm, and three arm lengths away from the pivot. The remaining panels demonstrate the principles governing the speed–accuracy trade-off using the target located at an angle of −*π*/3 relative to the horizontal. (*b*) At the slowest possible throw, the curve *ω*_0_(*ϕ*) is parallel to the *ϕ* axis, and the landing location is therefore insensitive to small variations in the release angle. At the other extreme, when the launch speed is infinitely large, throwing error is insensitive to variations in speed. Of the two limiting throws with infinite speed, the one with an infinite flight time will infinitely amplify even the smallest angle error. (*c*) The total squared error $\lambda^{2}=\lambda_{\phi }^{2}+\lambda_{\omega }^{2}$ is the result of a competition between a power-law decay in $\lambda_{\omega }^{2}$ versus a power-law rise in $\lambda_{\phi }^{2}$. The competition always leads to a local minimum in the total error. (*d*) The decay and rise have different exponents, with the decay of $\lambda_{\omega }^{2}$ always being faster than the rise in $\lambda_{\phi }^{2}$. Therefore, the local minimum in the total error exists for any linear weighted sum of the individual errors $\lambda_{\phi }^{2}$ and $\lambda_{\omega }^{2}$.

To understand this, we note that irrespective of the parameters being controlled by the throwing arm, the strategy for striking the centre of the target is specified by the curve *ω*_0_(*ϕ*) which has the same qualitative shape irrespective of the target’s location relative to the arm, below, at level or above, as seen from figure 4*a*. The thickened curves show *ω*_0_, and the inset shows the emerging speed–accuracy trade-off, for a target that is three arm-lengths away, and an angle *θ*_target_=−*π*/3 below the horizontal. Accuracy is given by

4.1 $$p(\omega )=\frac{1}{\lambda (\omega )}$$

and

4.2 $$\lambda^{2}=\lambda_{\phi }^{2}+\lambda_{\omega }^{2},$$

where the squared overall error λ^2^ is the sum of the squared errors $\lambda_{\phi }^{2}$ and $\lambda_{\omega }^{2}$, owing to variations in release angle and in release speed, respectively. Consider *ω*_0_(*ϕ*) for the overarm throw, as shown in figure 4*b*. At the slowest possible speed $\omega_{\min}$, the landing location is insensitive to small fluctuations *δϕ* in the release angle because the curve is parallel to the *ϕ* axis, and $\lambda_{\phi }^{2}(\omega_{\min})=0$. At the other extreme of ω→∞, speed fluctuations *δω* are parallel to the *ω* axis, and therefore $\lambda_{\omega }^{2}=0$. There are two limiting throwing angles where ω→∞, such that the flight time $t_{h}\to \infty$ for the release corresponding to the high curved throw, and *t*_*h*_→0 for the straight shot. With infinite flight time, $\lambda_{\phi }^{2}\to \infty$, and therefore *p*→0 as seen for the dotted lines in the inset of figure 4*a*. For the straight shot towards the target (inset of figure 4*c*), $\lambda_{\phi }^{2}=0$ at the minimum speed and $\lambda_{\phi }^{2}\to \lambda_{\infty }^{2}$ as ω→∞. On the other hand, $\lambda_{\omega }^{2}=\lambda_{\omega \min}^{2}$ at the minimum speed and $\lambda_{\omega }^{2}\to 0$ as ω→∞. The sum of these two has a local minimum, implying that there is a certain throwing speed that maximizes accuracy, and faster throws are always less accurate. The presence of a minimum for any linear weighted sum $\alpha \lambda_{\phi }^{2}+\beta \lambda_{\omega }^{2}$ depends only on the relative rate of decay of $\lambda_{\omega }^{2}$ being higher than the rate of growth of $\lambda_{\phi }^{2}$. In particular, it has no dependence on the weights *α* and *β* so long as *α*,*β*≠0. In other words, the relative noise level in the angle and speed has no impact on the speed–accuracy trade-off, because $\lambda_{\omega }^{2}$ decays faster than the growth in $\lambda_{\phi }^{2}$ (figure 4*d*) as given by (see the electronic supplementary material for derivation)

4.3 $$1-\frac{\lambda_{\phi }^{2}(\omega )}{\lambda_{\phi }^{2}(\infty )}∼\omega^{-2}$$

and

4.4 $$\lambda_{\omega }^{2}(\omega )∼\omega^{-6}.$$

The exponents are independent of the target’s location, and only depend on the nature of projectile flight under uniform gravity, as captured by the curve *ω*_0_(*ϕ*).

Our minimal model based on the error amplification properties of parabolic projectile flight exhibits the experimentally well-known trade-off between speed and accuracy in throwing [7]. Furthermore, it naturally justifies the qualitative observation that the most precise throw is slightly faster than the minimum throwing speed for hitting the target [5], independent of target geometry. This result emphasizes the importance of the physical task in characterizing speed–accuracy trade-offs, which are not likely to be just intrinsic properties of the motor system. Consistent with this, virtual reality throwing experiments in a non-uniform gravitational field by Sternad *et al.* [11] show that speed and accuracy do not always trade-off against each other, and whether they do depends on the task.

## 5. Implications

Our theory suggests a plausible mechanism for optimal strategies, and makes some simple predictions. We now follow some of its implications in the context of games that involve throwing, characterize the role of structured noise in the release parameters to determine how this plays out in determining optimal strategies, and conclude with some thoughts on learning the optimal strategy for throwing.

### 5.1. Dart throws

Dart throwing is a game that requires the accurate release of projectiles with a simple metric of performance that is easily quantified. Data on dart throwers from [3], show that the dart is released by human throwers at a speed of 5.8–6.7 m s^−1^ about 4–25 ms before the peak of a circular motion of the hand with a radius of 0.5–0.7 m. In the context of our model, we choose either the forearm or the whole arm as the natural length scale (see the electronic supplementary material, Scaling of experimental data). For a vertical target at the prescribed distance of 2.37 m in front of the thrower and 1.73 m above the ground, we calculate the optimal strategy *ϕ*^optim^ and *ω*^optim^=*ω*_0_(*ϕ*^optim^) that maximizes accuracy *p*(*ϕ*). The optimal dart throwing strategy is an overarm throw with an optimal release angle of 17–37^°^ before the arm becomes vertical, and a corresponding optimal speed of 5.1–5.5 m s^−1^. At this speed and release angle, the dart would be released 44–35 ms before the hand reaches the zenith. The best overarm throw is 7–20% more accurate than the best underarm throw, as found from the ratio of the accuracies *p*(*ϕ*^optim^). The overarm throwing strategy with a larger radius of curvature (0.8 m) and higher speed (5.5 m s^−1^) is the most accurate of all, consistent with observations. Our predictions are only weakly dependent on the choice of the length scale (see the electronic supplementary material), but are strongly dependent on the target geometry (see the electronic supplementary material). Similar calculations for basketball free throws, another sport with an emphasis on accuracy, also recover strategies that are consistent with observations (see the electronic supplementary material).

### 5.2. Effect of structured noise

We have so far assumed no covariance structure for the noise in the release angle *ϕ* and speed *ω*. In the linearized analysis, this is equivalent to having a uniform (circular) distribution of the errors in these variables with a variance (radius) *c* that is amplified through the projectile dynamics. Covariance structure to the input noise is manifest as an elliptic distribution of errors, say with semi-major axis *a* and semi-minor axis *b*. In order to compare two cases with equal noise, we constrain the area of the circle and ellipse to be the same, i.e. *ab*=*c*^2^. These distributions of initial conditions are propagated by the projectile dynamics to lead to an error *e*_cir_ or *e*_ell_, respectively. For small input noise, the radius of the input noise is amplified linearly by the singular value λ of the linearized projectile map *J*_err_ (figure 1*c*). Furthermore, using the fact that the smallest possible input error for the elliptic initial distribution, by definition of the semi-minor axis of the ellipse, is *b*, and *c*^2^=*ab*, we obtain *e*^2^_cir_=λ^2^*c*^2^=λ^2^*ab*, so that *e*^2^_ell_≥λ^2^*b*^2^. Therefore,

5.1 $$\frac{e_{\mathrm{ell}}}{e_{\mathrm{cir}}}\geq \sqrt{\frac{b}{a}}.$$

Even for strong covariance, i.e. b/a∼O(ϵ)≪1, the reduction in noise amplification is at best the square-root of the eccentricity of the covariance. Furthermore, to achieve this limit of noise reduction through covariation, the two release parameters have to covary exactly and compensate for each other’s amplification by the projectile dynamics, i.e. the major axis of the covariance ellipse has to exactly align with the null space of *J*_err_. If the major axis was misaligned by an angle *θ*, $e_{\mathrm{ell}}/e_{\mathrm{cir}}=\sqrt{b/a}\cos\theta +\sqrt{a/b}\sin\theta$. With strong covariance b/a∼O(ϵ) and a small misalignment of the covariance ellipse θ∼O(γ), γ≪1, we have $e_{\mathrm{ell}}/e_{\mathrm{cir}}=\sqrt{\epsilon }+\sqrt{\gamma }$. Therefore, the effect of a strong covariance is diminished, and the effect of a small misalignment is amplified. Structured noise, such as typical of human motor control [6,11,18,20], undoubtedly reduces the impact of noise on performance. However, we have shown here that for throwing, and in fact for any motor task where error amplification depends smoothly on the input parameters, using noise covariance to mitigate errors is a fragile strategy.

### 5.3. Planning under uncertainty

We conclude with a brief discussion of the role of uncertainty in planning before throwing, i.e. characterizing potential inaccuracies in the internal model of the projectile’s dynamics, characterized by a ‘planning uncertainty distribution’ *τ*(*ϕ*). This distribution models the uncertainty in the optimal strategy, which in turn is an outcome of uncertainty in the internal model of the projectile’s dynamics. The effective accuracy, following the definition of conditional probabilities, is given by

5.2 $$E_{\mathrm{over}}=\int_{\omega_{0}(\phi )<0}p(\phi \;|\;\tau )\tau (\phi )\;d\phi \;\mathrm{and}\;E_{\mathrm{under}}=\int_{\omega_{0}(\phi )\geq 0}p(\phi \;|\;\tau )\tau (\phi )\;d\phi .$$

When the internal model is uncertain we predict the optimal fraction of overarm and underarm throws as given by *f*_over_=*E*_over_/(*E*_over_+*E*_under_),*f*_under_=1−*f*_over_, a consequence of the fact that *E*_(•)_ is itself a random variable owing to uncertainty in the underlying model of the dynamics of the projectile, so that a minimal assumption is that *f*_(•)_ be proportional to *E*_(•)_, i.e. *p*(*ϕ*).

One natural limit of the planning probability distribution *τ*(*ϕ*) corresponds to perfect planning for an expert with zero uncertainty in the optimal overarm and underarm throw, i.e. $\tau (\phi )=\delta (\phi -\phi_{\mathrm{over}}^{\mathrm{optim}})+\delta (\phi -\phi_{\mathrm{under}}^{\mathrm{optim}})$ so that $E_{\left(∙\right)}=\max p_{\left(∙\right)}(\phi )$. The other limit is uniform planning with large uncertainty for a novice, i.e. *τ*(*ϕ*)=1 so that $E_{\left(∙\right)}=\int_{\left(∙\right)}p(\phi )\;d\phi$.

**Figure 5.**
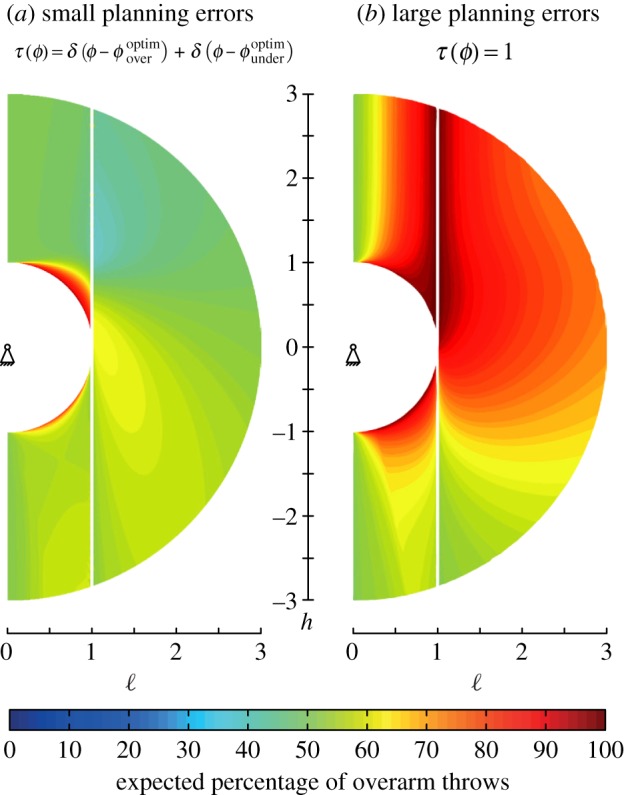
Predicted and measured fraction of overarm throws for a planar, horizontal target (like a bin). Colours correspond to expected fraction of overarm throws as a function of the height and distance of the target from the arm (of length unity). With zero planning errors (*a*), an underarm throw is strongly preferred for targets just outside arm length above the shoulder, while an overarm style is preferred for targets below the shoulder. By contrast, when planning errors are large (*b*), there is a strong preference for the overarm style almost independent of target location.

In figure 5*a*, we see that for no planning errors, an overarm throw is preferred for targets above the shoulder, but an underarm throw is preferred for targets below the shoulder. In figure 5*b*, we see that for large planning errors, an overarm throw is preferred for most target locations. Comparing this with figure 2*a*, we see that for a target below the shoulder, an overarm strategy is better, and also more forgiving of planning errors in *ϕ*. By contrast, comparing this with figure 2*b*, we see that this is consistent with the fact that accuracy *p* is strongly peaked for underarm throws towards a target above the shoulder but falls off rapidly, so that the underarm throw is optimal for small planning errors, but not robust to large planning errors. These predictions are also consistent with previous observations [2] that the preference of overarm versus underarm depends on the distance to the target (see the electronic supplementary material).

## 6. Discussion

The ability to throw fast and accurately is quintessentially human, and a seemingly complex task. Here, we have focused on the simplest physical problem of how errors in the release parameters are amplified by the parabolic trajectory of a thrown projectile to determine optimal strategies for throwing. Although throwing is a complicated motor task, the predictions of our model for overarm versus underarm throwing styles are consistent with extant experimental data that show a dependence of style on the target location as well as on planning uncertainty. Despite the absence of neural or physiological elements, our minimal model is consistent with a range of independent experiments on throwing: the speed–accuracy trade-off that is commonly observed in throwing [7], the optimal strategy (launch angle and speed) for throwing darts [3], the preference of overarm versus underarm throwing based on target location [2] and even the qualitative claim that the most precise throw is slightly faster than the minimum throwing speed for hitting the target [5]. Our work suggests that strategy and trade-offs are intimately related to the motor tasks that involve interactions with the environment, and are not just intrinsic properties of the neuromotor system. This should hardly be a surprise, since the system evolved and developed in a physical environment, but is a point worth emphasizing since it is all too often forgotten. We must look for the physical origins of the speed–accuracy trade-off that are central to the observed trade-offs in humans [5,7,9]. Given that throwing might have played a substantial role in our evolutionary past, some of our results on the trade-off between speed and precision, especially its dependence on the throwing style, may come to bear on the topic of human evolution.

Accurate throwing could serve as a testbed for understanding motor learning without the added difficulties of continuous feedback control present in tasks such as arm-pointing [14]. Indeed, our study naturally points to iterative approaches for learning that first execute a plan, observe errors or performance of the output, and use that to build an internal model. In a Bayesian framework, the planning distribution *τ*(*ϕ*) is the prior, *p*(*ϕ* | *τ*)*τ*(*ϕ*) is the observed posterior and the motor learning algorithm corresponds to the inference of the true model *p*(*ϕ*), i.e. an experiential understanding of Newtonian mechanics from repeated observations. Because the thrower’s performance depends on both *p* and *τ*, the thrower can employ *τ* as a probe to learn the dynamics of the task, i.e. *p*. Whether this is how we actually do learn about the physical world remains a question for the future.

## Supplementary Material

Supplementary notes

## Data accessibility

All detailed derivations are included in the electronic supplementary material. No other data were generated in this research.
