# Supplementary material for: Optimal strategies for throwing accurately
Source: R Soc Open Sci. 2017 Apr 26;4(4):170136. doi: 10.1098/rsos.170136 (PMC5414278; doi:10.1098/rsos.170136)
Supplement: Supplementary notes [file rsos170136supp1.pdf]

Online Supplement
Optimal strategies for throwing accurately

Madhusudhan Venkadesan[1,*], L. Mahadevan[2,3,†],

[1]Department of Mechanical Engineering & Materials Science, Yale University, New Haven, CT 06520, USA

[2]Department of Physics, and [3]Department of Organismic and Evolutionary Biology,

Paulson School of Engineering & Applied Sciences, Harvard University, Cambridge, MA 02138, USA

E-mail: ∗ m.venkadesan@yale.edu, † Lmahadev@g.harvard.edu

Contents

S1 Detailed derivation of equations 2

S2 Feasible arm angles 4

S3 Speed versus accuracy for vertical targets 6

S4 Generalization of the speed-accuracy trade-off 7

S4.1 Horizontal target 8

S4.2 Vertical target 10

S5 Scaling of experimental data 10

S6 Effect of planning uncertainty on strategy 11

S7 Basketball free throws 13

S8 Propagating distributions with non-infinitesimal variance 13

S9 Shooting: Zero arm length 16

S1 Detailed derivation of equations

Our model arm has independent control over the projectile's release angle (ϕ) and angular velocity (ω)/ For a drag-free point-like projectile moving in a uniform gravitational field, its trajectory $(x(t), y(t))$ as a function of time since release t is obtained by solving the initial value problem,

$$\ddot{x}(t) = 0,\ \dot{x}(0) = -\omega \sin\phi,\ y(0) = \cos\phi, \tag{S1.1a}$$

$$\ddot{y}(t) = -1,\ \dot{y}(0) = \omega\cos\phi,\ y(0) = \sin\phi, \tag{S1.1b}$$

where x and y are measured relative to the arm's pivot. We express lengths in units of the arm's length and time in appropriate units to set the acceleration due to gravity equal to one. Then, the solutions for the trajectory of the projectile that lands on a horizontally oriented, planar target (like a bin) located at a height h, and forward distance l are,

$$x(t,\phi,\omega) = \cos\phi - t\omega\sin\phi = l \tag{S1.2a}$$

$$y(t,\phi,\omega) = \sin\phi + t\omega\cos\phi - \frac{1}{2}t^2 = h \tag{S1.2b}$$

We note that the calculations proceed in an analogous manner for other target geometries, with modifications for the definition of error as shown in figure S1.

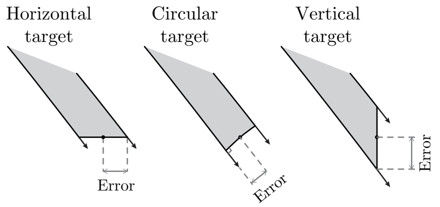

Figure S1. The definitions of landing error for three different target geometries, the horizontal target that was extensively analyzed in the main paper , a circular target where error is defined as the distance of nearest approach, and a vertical target the error is defined along a vertical axis. The gray band depicts a set of trajectories approaching the target.

Solving equation [**S1.2b**] for t, and using the condition $\dot{y}(t) = \omega\cos\phi - t \leq 0$ for the projectile to strike the upper face of the target, we obtain a solution surface $t_h(\phi,\omega)$. On substituting $t = t_h(\phi,\omega)$ in equation [**S1.2a**] we obtain the equation for the horizontal landing location of the projectile when it strikes the plane of the horizontal target as,

$$t_h(\phi,\omega) = \omega\cos\phi + \sqrt{\omega^2\cos^2\phi - 2(h-\sin\phi)} \tag{S1.3a}$$

$$x_h(\phi,\omega) = \cos\phi - \omega\sin\phi\left(\omega\cos\phi + \sqrt{\omega^2\cos^2\phi - 2(h-\sin\phi)}\right). \tag{S1.3b}$$

We require $\omega^2 \geq 2(h - \sin\phi)/\cos^2\phi$ for a solution to exist, i.e. a minimum launch velocity is needed to reach the target plane when $h \geq \sin\phi$. For some target positions and arm postures, it is impossible to hit the target because no real solutions exist for the minimum launch velocity. We enumerate all feasible arm angles in Supplement S2.

We set $x_h(\phi, \omega) = l$ in equation [**S1.3b**] for a target at a distance l to obtain a one parameter family of solutions for exactly striking a point target, as given by

$$\omega_0(\phi) = \frac{\cos\phi - l}{\sin\phi\sqrt{\frac{2}{\sin\phi}\left(1 - l\cos\phi - h\sin\phi\right)}} \,. \tag{S1.4}$$

When the release parameters (ϕ, ω) deviate from the one-dimensional solution curve $(\phi, \omega_0(\phi))$, the projectile misses the target leading to an error $\delta x_h = x_h(\phi, \omega) - l$. To quantify the amplification of small launch errors, we linearize $x_h(\phi, \omega)$ in the neighborhood of the curve $(\phi, \omega_0(\phi))$ or $(\omega, \omega_0^{-1}(\omega))$ depending upon whether we want to parameterize error amplification by the launch angle or speed. We then obtain the relation between the 'input errors' $(\delta\phi\ \delta\omega)^{\mathrm{T}}$ and the 'output/target error' δx_h given by

$$\delta x_h(\phi, \omega) \approx J_{\text{err}} \begin{pmatrix} \delta\phi \\ \delta\omega \end{pmatrix}, \tag{S1.5a}$$

$$\text{where } J_{\text{err}} = \begin{cases} J_{\text{err}}(\phi) = \begin{pmatrix} \frac{\partial x_h}{\partial \phi} & \frac{\partial x_h}{\partial \omega} \end{pmatrix}\Big|_{\omega=\omega_0(\phi)}, \text{ or} \\ J_{\text{err}}(\omega) = \begin{pmatrix} \frac{\partial x_h}{\partial \phi} & \frac{\partial x_h}{\partial \omega} \end{pmatrix}\Big|_{\phi=\omega_0^{-1}(\omega)} \end{cases}. \tag{S1.5b}$$

The amplification of these errors is quantified by the only non-zero singular value λ of J_{err}. For illustration of computing the singular value, we choose the parameterization of error amplification as a function of ϕ, where the error Jacobian is computed in the neighborhood of $\omega_0(\phi)$ according to,

$$J_{\text{err}}(\phi) = \begin{pmatrix} \lambda_\phi & \lambda\omega \end{pmatrix}, \tag{S1.6a}$$

$$\lambda_\phi = \frac{\partial x_h}{\partial \phi}\Big|_{\phi, \omega_0(\phi)} = \frac{4hl\cos\phi - 4(h + l\cot\phi) + \csc\phi\left((2l^2 - 1)\cos 2\phi + 3\right)}{4h\sin\phi + 2l\cos\phi + \cos 2\phi - 3} \tag{S1.6b}$$

$$\lambda_\omega = \frac{\partial x_h}{\partial \omega}\Big|_{\phi, \omega_0(\phi)} = \frac{4\sqrt{2}\sin^2\phi(\csc\phi - l\cot\phi - h)^{\frac{3}{2}}}{4h\sin(\phi) + 2l\cos(\phi) + \cos(2\phi) - 3}. \tag{S1.6c}$$

The only non-zero singular value $\lambda(\phi)$ of $J_{\text{err}}(\phi)$ is given by,

$$\lambda^2(\phi) = \lambda_\phi^2 + \lambda_\omega^2 \tag{S1.7}$$

This singular value has a geometric interpretation, as seen from comparing equa-

tion [**S1.5a**] to the lowest order Taylor series expansion of $(\delta x_h)^2$,

$$(\delta x_h)^2 = \begin{pmatrix}\delta\phi \\ \delta\omega\end{pmatrix}^{\mathrm{T}} J_{\mathrm{err}}^{\mathrm{T}} J_{\mathrm{err}} \begin{pmatrix}\delta\phi \\ \delta\omega\end{pmatrix} = \frac{1}{2}\begin{pmatrix}\delta\phi \\ \delta\omega\end{pmatrix}^{\mathrm{T}} H_{\mathrm{err}} \begin{pmatrix}\delta\phi \\ \delta\omega\end{pmatrix}. \tag{S1.8}$$

$2\lambda^2$ is the largest eigenvalue of the Hessian (H_{err}) of the output variance (δx_h^2) and thus the maximal principal curvature of the surface $\delta x_h^2(\phi, \omega_0(\phi)) = 0$. The error varies most weakly along the curve $(\phi, \omega_0(\phi))$ and strongly in the orthogonal direction to that curve.

S2 Feasible arm angles

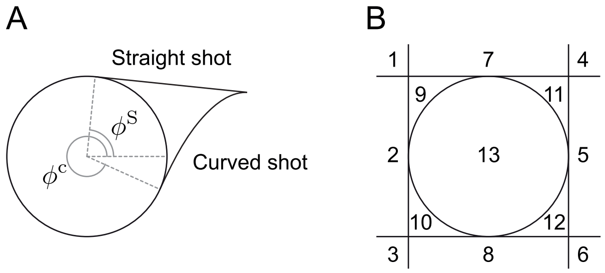

Figure S2. The individual cases for identifying the feasible arm angles in order to be able to strike a planar target. **a.** Two qualitatively different shallow shots to hit the target depending on whether the target is above or below the point of release. **b.** Enumeration of different regions of target location where domains of ϕ are calculated for $x_h(\phi, \omega_0(\phi))$ to be a real number.

For some target positions and arm postures, it is impossible to hit the target because no real solutions exist for the minimum launch velocity. We enumerate all feasible arm angles in this section. The ball release is always a tangent to the circle defined by the arm length. These equations are then obtained by solving for tangency of various parabolic and straight flight paths corresponding to the numbered domains in figure S2. These domains are used to calculate feasible arm angles at release so that there exists a speed of release that can reach locations within the domain.

To calculate the arm angles where it is possible to hit the target, we first observe that there are two qualitatively different shallow shots depending on the height of the target relative to the release point (Figure S2a). We refer to the straight-line shot with infinite velocity by $\phi^{\mathrm{s}}_{(.)}$ and the curved shallow shot which is a parabola passing through the target with its apex at the target, and tangential to the unit circle by $\phi^{\mathrm{c}}_{(.)}$. The subscript will

denote the throwing style (‘u’ or ‘o’). These angles are given by,

$$c(\alpha) = (1+l)\tan^4\frac{\alpha}{2} - 4h\tan^3\frac{\alpha}{2} + 6\tan^2\frac{\alpha}{2} - 4h\tan\frac{\alpha}{2} + (1-l) \tag{S2.1a}$$

$$\phi_{\mathrm{o}}^{\mathrm{c}} = \begin{cases} \max\alpha & \text{where } \alpha \text{ is a real root of } c(\alpha)\text{if } l<1 \\ \min\alpha & \text{if } l>1 \end{cases} \tag{S2.1b}$$

$$\phi_{\mathrm{u}}^{\mathrm{c}} = \begin{cases} \min\alpha & \text{if } l<1 \\ \max\alpha & \text{if } l>1 \end{cases} \tag{S2.1c}$$

$$\phi_{\mathrm{o}}^{\mathrm{s}} = \arctan 2(h,l) + \arccos\frac{1}{d} \tag{S2.1d}$$

$$\phi_{\mathrm{u}}^{\mathrm{s}} = \arctan 2(h,l) - \arccos\frac{1}{d} \tag{S2.1e}$$

$$\arctan 2(h,l) = \arg(l+ih) \tag{S2.1f}$$

$$d = \sqrt{l^2+h^2} \tag{S2.1g}$$

[**S2.1a**] has only two real roots in the interval $[0, 2\pi]$. The domains of feasible ϕ so that $x_h(\phi, \omega_0(\phi))$ is a real number are given by,

$1: l < -1, h \geq 1$	$\Phi = (0, \phi_{\mathrm{u}}^{\mathrm{c}}] \cup (\pi, \phi_{\mathrm{o}}^{\mathrm{c}}]$
$2: l < -1, \lvert h\rvert < 1$	$\Phi = (0, \phi_{\mathrm{u}}^{\mathrm{s}}) \cup (\pi, \phi_{\mathrm{o}}^{\mathrm{c}}]$
$3: l < -1, h \leq -1$	$\Phi = (0, \phi_{\mathrm{u}}^{\mathrm{s}}) \cup (\pi, \phi_{\mathrm{o}}^{\mathrm{s}})$
$4: l > 1, h \geq 1$	$\Phi = [\phi_{\mathrm{u}}^{\mathrm{c}}, 2\pi) \cup [\phi_{\mathrm{o}}^{\mathrm{c}}, \pi)$
$5: l > 1, \lvert h\rvert < 1$	$\Phi = [\phi_{\mathrm{u}}^{\mathrm{c}}, 2\pi) \cup (\phi_{\mathrm{o}}^{\mathrm{s}}, \pi)$
$6: l > 1, h \leq -1$	$\Phi = (\phi_{\mathrm{u}}^{\mathrm{s}}, 2\pi) \cup (\phi_{\mathrm{o}}^{\mathrm{s}}, \pi)$
$7: \lvert l\rvert < 1, h > 1$	$\Phi = (0, \phi_{\mathrm{u}}^{\mathrm{c}}] \cup [\phi_{\mathrm{o}}^{\mathrm{c}}, \pi)$
$8: \lvert l\rvert < 1, h < -1$	$\Phi = ((0, \psi] \cup (\phi_{\mathrm{u}}^{\mathrm{s}}, 2\pi - \psi]) \cup ([\psi, \pi) \cup [2\pi - \psi, \phi_{\mathrm{o}}^{\mathrm{s}}))$
$9: -1 < l < 0, 0 < h \leq 1$	$\Phi = (0, \phi_{\mathrm{u}}^{\mathrm{s}}) \cup [\phi_{\mathrm{o}}^{\mathrm{c}}, \pi)$
$10: -1 < l < 0, -1 \leq h < 0$	$\Phi = ((0, \psi] \cup (\phi_{\mathrm{u}}^{\mathrm{s}}, 2\pi - \psi]) \cup ([\psi, \pi) \cup [2\pi - \psi, \phi_{\mathrm{o}}^{\mathrm{c}}])$
$11: 0 < l < 1, 0 < h \leq 1$	$\Phi = (0, \phi_{\mathrm{u}}^{\mathrm{c}}] \cup (\phi_{\mathrm{o}}^{\mathrm{s}}, \pi)$
$12: 0 < l < 1, -1 \leq h < 0$	$\Phi = ((0, \psi] \cup [\phi_{\mathrm{u}}^{\mathrm{c}}, 2\pi - \psi]) \cup ([\psi, \pi) \cup [2\pi - \psi, \phi_{\mathrm{o}}^{\mathrm{s}}))$
$13: l^2 + h^2 \leq 1$	$\Phi = (0, \psi] \cup [\psi, \pi)$

where $\psi = \arccos(l)$, and the domains are listed in the format (underarm) $\cup$ (overarm).

S3 Speed versus accuracy for vertical targets

Using a modified definition of error for a vertical target as shown in figure S1, and the same method of derivation as shown in section S1, we find the accuracy p as a function of launch speed ω for several targets. A speed-accuracy trade-off, similar to that of a horizontal target, is observed for the vertical target so long as it is located at or above the shoulder height, as seen in figure S3. For targets below shoulder height, we find faster underarm throws to be more accurate. This reversal of the speed-accuracy trade-off is however, true only for a small region of targets near the arm. For vertical targets more than 3.5 arm lengths below the shoulder pivot or horizontally away from the shoulder, the usual speed-accuracy trade-off is observed. Casual observations of striking the wicket in cricket from proximal locations such as the wicket keeper's action are consistent with our calculations that predict that a fast underarm throw is the most accurate strategy for a vertical target like the wickets that is typically less than 3m away from the thrower.

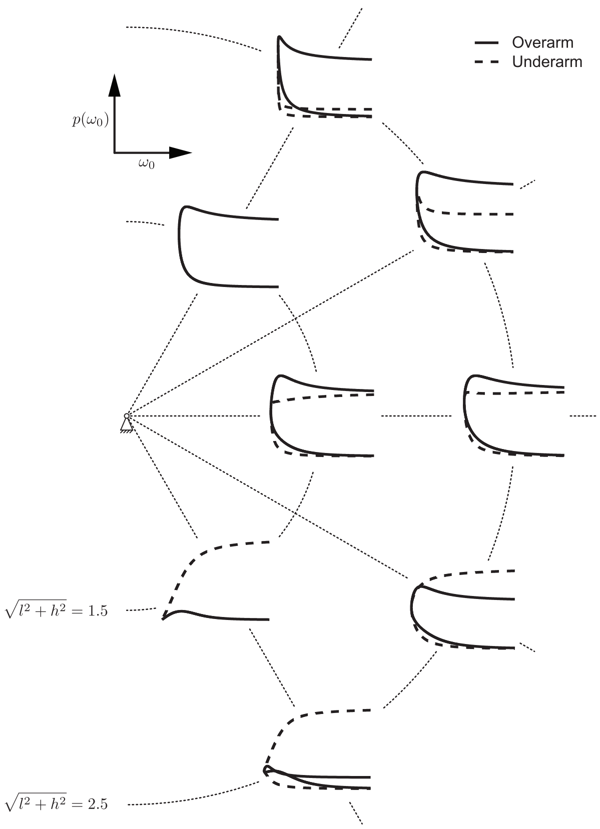

Figure S3. Accuracy as a function of speed for several vertically oriented targets.

S4 Generalization of the speed-accuracy trade-off

The error in the projectile's landing location e, is defined based on the target geometry (see figure S1). For example, $e = x_h - l$ for a horizontally oriented target, and $e = y_l - h$ for a vertically oriented target. The accuracy p is given by,

$$p = \frac{1}{\lambda}, \tag{S4.1a}$$

$$\tag{S4.1b}$$

following equations [**S1.7**] for the definition of λ, so that,

$$\lambda^2 = \lambda_\phi^2 + \lambda_\omega^2, \tag{S4.2a}$$

$$\text{where } \lambda_\phi = \frac{\partial e}{\partial \phi},\ \lambda_\omega = \frac{\partial e}{\partial \omega}. \tag{S4.2b}$$

For a horizontal target, i.e. $e = x_h - l$, the expressions for λ_ϕ and λ_ω are given by equations [**S1.6**]. The dependence of λ_ϕ^2 and λ_ω^2 on the speed ω, are therefore central to characterize accuracy as a function of throwing speed. A projectile launched with a speed $\omega = \omega_0(\phi)$, as given by equation [**S1.4**] will strike the target with zero error. The non-invertible nature of $\omega_0(\phi)$ implies that there are four distinct throws, launched at four different launch angles, which strike a target at its center. For our analysis, we consider the limit $\omega \to \infty$. With increasing speed, the flight time $t_f \to 0$ for the two straight shots (overarm and underarm). For the curved shots however, higher speeds imply longer flight times, and $t_f \to \infty$. Therefore, the landing location for both the curved shots are infinitely sensitive to the launch angle in the limit of the throwing speed becoming infinite. We will therefore only consider the shallow shots for the remainder of this analysis. Also, we consider only non-trivial targets where simply dropping the projectile with zero speed is not a solution, i.e. $l > 1$, and $l^2 + h^2 > 1$. The straight shot for the overarm and underarm throw correspond to a launching angle of

$$\phi_{\mathrm{o}}^{\mathrm{s}} = \arctan 2(h, l) + \arccos \frac{1}{d}, \text{ and} \tag{S4.3a}$$

$$\phi_{\mathrm{u}}^{\mathrm{s}} = \arctan 2(h, l) - \arccos \frac{1}{d}, \tag{S4.3b}$$

respectively. Note that the straight overarm and underarm shot are impossible for horizontal targets with $h > 1$ and $h > -1$, respectively. This is because we consider horizontal targets like bins or basketball hoops, where hitting the target from below is disallowed.

From equation [**S1.4**], the leading-order asymptotic expansion of $\omega_0(\phi)$ as $\phi \to \phi_{\mathrm{o/u}}^{\mathrm{s}}$,

and denoting overarm or underarm by o/u, is given by,

$$\omega^2 = c_{\mathrm{o/u}} \left(\phi - \phi^{\mathrm{s}}_{\mathrm{o/u}}\right), \tag{S4.4a}$$

$$c_{\mathrm{o/u}} = \frac{l(l^2+h^2-1) \pm h\sqrt{l^2+h^2-1}}{2(l^2+h^2)} > 0. \tag{S4.4b}$$

This expansion of ω for large speeds, i.e. as $\phi \to \phi^{\mathrm{s}}_{\mathrm{o/u}}$ will be used to find asymptotic expansions for λ_ϕ and λ_ω.

S4.1 Horizontal target

Consider targets with $h < 1$ for the overarm style, or $h < -1$ for the underarm style. Using equation [**S1.6b**] in the limit of the straight shot $\omega \to \infty$, i.e. $\phi \to \phi^{\mathrm{s}}_{\mathrm{o/u}}$, the leading order asymptotic expansion of the sensitivity to release angle is given by,

$$\lambda_\phi^2 = \lambda_{\phi\infty}^2 + m_\phi(\phi - \phi^{s}_{\mathrm{o/u}}) + \mathcal{O}\left((\phi - \phi^{s}_{\mathrm{o/u}})^2\right), \tag{S4.5}$$

with constants $\lambda_{\phi\infty}$ and m_ϕ given by,

$$\lambda_{\phi\infty} = \lambda_\phi\left(\phi^{\mathrm{s}}_{\mathrm{o/u}}\right), \tag{S4.6a}$$

$$m_\phi = \left.\frac{\partial \lambda_\phi^2}{\partial \phi}\right|_{\phi^{\mathrm{s}}_{\mathrm{o/u}}} < 0. \tag{S4.6b}$$

Using equation [**S4.4a**] in equation [**S4.5**] to rewrite the expansion in terms of the launch speed ω, and noting that $m_\phi < 0$,

$$\frac{\lambda_\phi^2}{\lambda_{\phi\infty}^2} = 1 - \frac{|m_\phi|\, c_{\mathrm{o/u}}}{\lambda_{\phi\infty}^2}\omega^{-2} + \mathcal{O}\left(\omega^{-3}\right), \tag{S4.7a}$$

$$\implies 1 - \frac{\lambda_\phi^2}{\lambda_{\phi\infty}^2} \sim \omega^{-2}. \tag{S4.7b}$$

Therefore, for overarm and underarm horizontal throws of increasing speeds, the error λ_ϕ saturates towards $\lambda_{\phi\infty}$ with an exponent of 2.

Using equation [**S1.6c**], the leading order asymptotic expansion of the sensitivity to release speed is given by,

$$\lambda_\omega^2 = m_\omega(\phi - \phi^{\mathrm{s}}_{\mathrm{o/u}})^3 + \mathcal{O}\left((\phi - \phi^{\mathrm{s}}_{\mathrm{o/u}})^4\right), \tag{S4.8a}$$

$$m_\omega = \left.\frac{1}{3!}\frac{\partial^3 \lambda_\omega^2}{\partial \phi^3}\right|_{\phi^{\mathrm{s}}_{\mathrm{o/u}}} > 0. \tag{S4.8b}$$

Using equation [**S4.4a**] in equation [**S4.8a**] we rewrite the expansion in terms of the launch speed ω as,

$$\lambda_\omega^2 = m_\omega c_{\mathrm{o/u}} \omega^{-6} + \mathcal{O}\left(\omega^{-7}\right), \tag{S4.9a}$$

$$\implies \lambda_\omega^2 \sim \omega^{-6}. \tag{S4.9b}$$

Suppose the noise level in release angle and speed are not equal, and parameterized by the ratio of the noise level in speed versus angle $k = \delta\omega/\delta\phi$. The combined error λ due to both angle and speed sensitivities is then given by,

$$\lambda^2 = \lambda_\phi^2 + k^2 \lambda_\omega^2 \tag{S4.10a}$$

$$\implies \frac{\lambda^2}{\lambda_{\phi\infty}^2} = 1 - \frac{|m_\phi|\, c}{\lambda_{\phi\infty}^2}\omega^{-2} + \frac{k^2 m_\omega c}{\lambda_{\phi\infty}^2}\omega^{-6}. \tag{S4.10b}$$

The error λ^2 has a local (and global) minimum at $\omega = \pm\omega^*$ for the underarm and overarm style, where

$$\omega^* = \sqrt{k}\left(\frac{3m_\omega}{|m_\phi|}\right)^{1/4}, \tag{S4.11a}$$

$$\lambda(\omega^*)^2 = \lambda_{\phi\infty}^2 - \frac{2}{3\sqrt{3}}\frac{c\,|m_\phi|^{3/2}}{k\sqrt{m_\omega}}. \tag{S4.11b}$$

For speeds higher than the optimal speed, where the asymptotic results are increasingly accurate, the squared error grows monotonically. However, higher relative levels of noise in release speed implies a higher optimal speed, and a weaker trade-off between speed and accuracy. Nevertheless, and independent of the release speed, increasing the throwing speed beyond the optimal value ω^* always implies growing error, and therefore decreasing accuracy. This qualitative result is independent of the relative noise levels, the chosen length and time scales for the non-dimensional analysis, or on the parameters being controlled by the throwing arm.

All the other targets, $h > 1$ with the overarm style or $h > -1$ with the underarm style, can only be reached by a curved shot (overarm or underarm). Because the flight time grows to infinity with higher speeds for the curved shot, the sensitivity to the release angle goes to infinity as well, i.e. $\lim_{\omega\to\infty} \lambda_\phi \to \infty$. For these curved shots, the two sensitivities scale as,

$$\lambda_\phi^2 \sim \omega^4, \tag{S4.12a}$$

$$\lambda_\omega^2 \sim \omega^{-2} \tag{S4.12b}$$

This implies more severe speed-accuracy trade-off for these targets because the total error

suffers an unbounded increase with speed.

S4.2 Vertical target

For the overarm throwing style, and for all possible targets, the scaling of error with speed is identical to the analysis presented for the horizontal target in equations [**S4.7**] and [**S4.9**].

Using an underarm style, however, a qualitative difference arises for λ_ϕ^2. In contrast to equation [**S4.7**], we find,

$$\frac{\lambda_\phi^2}{\lambda_{\phi\infty}^2} - 1 \sim \omega^{-2}. \tag{S4.13}$$

Therefore, the sensitivity λ_ϕ^2 attains a local maximum at an intermediate throwing speed and subsequently decays to its asymptotic value. Therefore, both the sensitivities λ_ϕ^2 and λ_ω^2 have decreasing values with increasing speed, beyond a critical value. However, whether this is an optimal speed that minimizes error depends on the ratio,

$$\chi = \frac{\lim\limits_{\omega\to\infty} \lambda_\phi^2}{\lim\limits_{\omega\to\omega_{\min}} \lambda_\omega^2}. \tag{S4.14}$$

The ratio χ depends solely on the target location. When $\delta\omega/\delta\phi = k$, and $\chi > k^2$, the optimal speed is close to the minimum feasible speed $\omega_{\min}$, and faster underarm throws are less accurate. When $\chi < k^2$, faster underarm throws are more accurate. Figure S4 shows the dependence of χ on the target location. For $k = 1$, the solid red curve separates the region of targets with the usual speed-accuracy trade-off for the underarm throw, from the ‘faster is more accurate’ region.

S5 Scaling of experimental data

In order to compare experimental data against the dimensionless predictions of our model, we use published anthropometric data [1, 4]. We calculate a histogram of dimensionless parameters l and h for the dimensional distance L and height H of the target using the formulas $l = L/R$ and $h = (H - S)/R$, where R is the arm length, and S is the shoulder height above ground. We estimate R and S, normalized by total height, from previously published anthropometric data [1], namely, $S = 0.86$(Total Height) and $R = 0.54S$. Data on overall height for Americans, categorized by age, gender and year of measurement, are available from Ogden et al. [4].

The standard rules of the World Darts Federation stipulate that the center of the dart board (the bull) is 2.37m in front (L), and 1.73m above the ground (H). We normalize

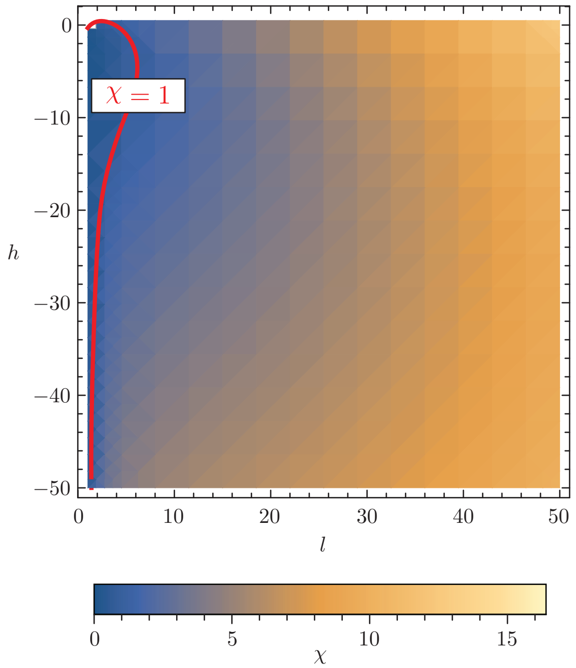

Figure S4. Dependence of the ratio χ on the target location. The value of χ relative to k^2, square of the ratio of velocity to angle noise, determines whether the underarm style exhibits the typical speed-accuracy trade-off or not. When $\chi < k^2$, faster underarm throws are more accurate. Therefore, for large noise in release velocity relative to release angle, faster underarm throws are more accurate than slower ones for almost all targets.

the dart board's location relative to the shoulder using the procedure outlined above.

S6 Effect of planning uncertainty on strategy

We compare against the data of Westergaard et al. [8], who used repeated throws in humans to estimate the proportion of overarm throws. The Westergaard et al. [8] experiment consisted of throwing a ball into a 0.8 m tall bin (H) for two targets — one 'near' ($L_1 = 3$ m) and another 'far' ($L_2 = 6$ m). For the age distribution of subjects that participated in [8], and using the procedure outlined in section S5, we generate histograms for the expected fraction of overarm throws in both the large and small planning error limits. In figure S5, we show the result of our calculations using an ellipse or a square box, corresponding to the results of the calculation for a circular to a horizontal target. The mean location of the symbol is the mean of the calculations, allowing for body size variations, and the height of the symbol is the range of calculations. The open symbols □ and ○ correspond to the estimated fraction of overarm throws with zero planning uncertainty, while the solid symbols ■ and ● are the estimated fraction of overarm throws

with maximal planning uncertainty. The + symbols are the experimentally measured means [8]. The gray bar shows the complete experimental range, lumping both targets, and corresponds well with the total range that we would predict.

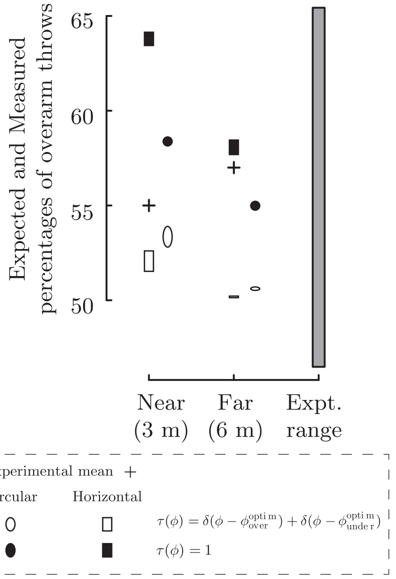

Figure S5. The result of our calculations in comparison with data. The symbols of an ellipse (○ for zero planning uncertainty and ● for maximal planning uncertainty) or a square box (□ for zero planning uncertainty and ■ for maximal planning uncertainty), correspond to the results of the calculation for a circular or a horizontal target, respectively. Because the experimental data (+ symbol) were measured for a horizontal bin [8], we expect the calculations for a horizontal target (□, ■) to correspond well with the experimental mean, and the calculations for a circular target (○, ●) to not match the data. The mean location of the symbol is the mean of the calculations, allowing for body size variations, and the height of the symbol is the range of calculations. For the closer target, the experimental data correspond to the predictions with zero planning uncertainty. For the target that is further away, the predictions with large planning uncertainty are a better predictor of the fraction of overarm throws that are used.

For two targets corresponding to bins at 3m and 6m, figure S5 (square symbols □ and ■) shows an overlay of our predictions on the experimental data for observed fraction of overarm throws [8]. Because visual estimates are likely more accurate for the closer bin, we expect smaller planning errors for the nearer target. Thus throwing into the bin at 3m corresponds better to $\tau(\phi) = \delta(\phi - \phi^{\text{optim}}_{\text{over}}) + \delta(\phi - \phi^{\text{optim}}_{\text{under}})$ while throwing into the bin at 6m corresponds better to $\tau(\phi) = 1$.

S7 Basketball free throws

The implications of our analysis to ball games suggests some comparisons to observations and data on free throws in basketball. Most professional players such as those in the American National Basketball Association (NBA) use an overarm throw for free throws. Using the average height for an NBA player, the dimensionless distance and height of the basket from the player for a free throw are 5.05 and 1.38, respectively. Our model predicts that for zero and uniform (arbitrary) planning uncertainty [**11b** in main text], f_{over}, the fraction of overarm throws, should be 49% and 64%, respectively. The numbers are similar for an average American male as well ($f_{\text{over}} = 50\%$ and 62% for zero and infinite planning uncertainty, respectively). Therefore, our model predicts that an overarm throw is a better choice for larger noise. However, if one could train to reduce sensorimotor noise, then the underarm throw is marginally better for an average NBA player. This relates to the observation that although the underarm throw is seldom used in professional basketball, one of the most proficient free throw shooter with a 90% success rate, Rick Barry, used the underarm style [5, 6]. However, given that our model predicts such a small margin of preference for the underarm style, it is likely that one of the many neuromechanical or psychological factors we did not include in our model will influence the choice of throwing style. Okubo and Hubbard [5] also find that the underarm throw is marginally superior for the 'typical' free throw in basketball by using a model with three-dimensional dynamics of a basketball with spin, non-ideal collisions with the backboard and rim, rolling on the rim, and so on. We arrive at a similar finding using only projectile dynamics with our relatively simpler model.

S8 Propagating distributions with non-infinitesimal variance

We next relax assumptions of the linearized analyses by introducing finite noise at the input side using a joint probability density function (PDF) $f_{[\phi,\omega]}$ associated with the noise in ϕ and ω. The horizontal distance x_h at which the projectile lands is given by the non-invertible, surjective function $x_h(\phi, \omega)$, which transforms $f_{[\phi,\omega]}$ into the PDF f_{x_h} associated with x_h. For a fixed ϕ, $f_{[\phi,\omega]}(\phi, \omega_0(\phi, l))$ is transformed into $f_{x_h}(l)$ by the Jacobian of ω_0 with respect to l. However, for a given target at distance l and height h, there is a curve of release parameters $(\phi, \omega_0(\phi, l))$ for accurately striking the target. Therefore, integrating

over this solution curve and using [**5** in main text] for ω_0, we find f_{x_h} as,

$$f_{x_h}(l) = \frac{\int\limits_{\Phi} f_{[\phi,\omega]}(\phi', \omega_0(\phi', l)) \left| \frac{\partial \omega_0(\phi', l)}{\partial l} \right| d\phi'}{\int\limits_{-\infty}^{\infty} \int\limits_{\Phi} f_{[\phi,\omega]}(\phi', \omega_0(\phi', l)) \left| \frac{\partial \omega_0(\phi', l)}{\partial l} \right| d\phi' \, dl} \tag{S8.1a}$$

$$\frac{\partial \omega_0(\phi, l)}{\partial l} = \frac{\csc^2 \phi (4h \sin\phi + 2l \cos\phi + \cos 2\phi - 3)}{4\sqrt{2}(\cot\phi + \csc\phi - h - l)^{3/2}} \tag{S8.1b}$$

where $\Phi = \{\phi : x_h(\phi, \omega_0(\phi, l)) = l\}$ is typically disjoint and depends on the location of the target as enumerated in S2. To ensure that f_{x_h} is a probability density with total area equal to one, we normalize [**S8.1a**] [1].

To follow the implications of the fully nonlinear calculation for error amplification, we choose the simple example of throwing a projectile into a bin. For this example, we assume uncorrelated noise in ϕ and ω, $f_{[\phi,\omega]} = f_\phi f_\omega$, where f_ϕ is a von Mises distribution. Because ϕ is a periodic variable, we use a von Mises distribution [3, 7], which is the circular analogue of a Gaussian distribution. The PDF of a von Mises distribution with mean μ and concentration κ ($1/\kappa$ is analogous to the variance of a normal distribution) is given by,

$$v(x, \mu, \kappa) = \frac{e^{\kappa \cos(x-\mu)}}{2\pi I_0(\kappa)} \tag{S8.2}$$

where $I_0(\kappa)$ is the modified Bessel function of order 0. For f_ω, we use a one-sided truncated Gaussian in order to restrict ω to a single throwing style at a time, i.e. $\omega \geq 0$ or $\omega < 0$ for underarm and overarm, respectively. The PDF of a truncated Gaussian variable x with mean μ and standard deviation σ that is truncated at a and b is given by,

$$g(x, \mu, \sigma, a, b) = \begin{cases} \sqrt{\frac{2}{\pi}} \frac{\frac{1}{\sigma} e^{-\frac{(x-\mu)^2}{2\sigma^2}}}{\left(\text{erf}\left(\frac{b-\mu}{\sigma\sqrt{2}}\right) - \text{erf}\left(\frac{a-\mu}{\sigma\sqrt{2}}\right)\right)} & \text{if } a \leq x \leq b \\ 0 & \text{Otherwise} \end{cases} \tag{S8.3}$$

where erf is the Gauss error function. Therefore, the distributions we use for numerical examples are,

$$f_{[\phi,\omega]}(\phi', \omega') = f_\phi(\phi') \, f_\omega(\omega') \tag{S8.4a}$$

$$f_\phi(\phi') = v(\phi', \phi_{(\cdot)}^{\text{optim}}, 1/\sigma_\phi^2), \; (\cdot) = \text{over or under} \tag{S8.4b}$$

$$f_\omega(\omega') = \begin{cases} g(\omega', \omega_0(\phi_{\text{over}}^{\text{optim}}), \sigma_\omega, -\infty, 0) & \text{for overarm} \\ g(\omega', \omega_0(\phi_{\text{under}}^{\text{optim}}), \sigma_\omega, 0, \infty) & \text{for underarm} \end{cases} \tag{S8.4c}$$

[1] There is ‘leakage’ because $x_h(\phi, \omega)$ is not a real number for some combinations (ϕ, ω) when the projectile fails to reach the plane of the target, and thus normalization becomes necessary.

where σ is the standard deviation, and ϕ^{optim} denotes the optimal throwing angle such that the linear amplification $\lambda(\phi^{\text{optim}})$ is the minimum for the chosen throwing style [**S1.7**].

We now perform linear and nonlinear analyses for a specific numerical example, with $l = 2$, $h = -1$. For this bin, we use the linear error amplification λ to find the angle ϕ and speed ω for the optimal overarm and underarm throws. The input distributions for the nonlinear calculation are centred at the optimal release parameters found from this linear analysis. The variance of the distributions are arbitrarily chosen, but are equal for both throwing styles. Table S1 lists the numerical values used in this example. Dimensional values are shown for a one meter long arm.

Table S1. Parameters values used in nonlinear calculations for bin at $l = 2$, $h = -1$. Dimensional estimates for an arm length of 1m.

Overarm				Underarm			
f_ϕ		f_ω		f_ϕ		f_ω	
$\phi^{\text{optim}}_{\text{over}}$	σ_ϕ	$\omega_0(\phi^{\text{optim}}_{\text{over}})$	σ_ω	$\phi^{\text{optim}}_{\text{under}}$	σ_ϕ	$\omega_0(\phi^{\text{optim}}_{\text{under}})$	σ_ω
1.08	0.09	-1.19	0.18	5.72	0.09	1.01	0.18
62°	5°	3.72 m/s<br>13.4 km/hr	0.56 m/s<br>2 km/hr	-33°	5°	3.17 m/s<br>11.4 km/hr	0.56 m/s<br>2 km/hr

Our numerical solutions show that the underarm throw amplifies errors more than the overarm throw (solid curves in Figure S6), in agreement with the linear amplification λ. Quantitatively as well, the ratio of overarm to underarm linear amplification is similar to the ratio of σ, the standard deviation of the output distribution f_{x_h} (see Table S2). However, the nonlinear calculation calculation shows that the output distribution for an underarm throw is skewed (comparison with dashed curve in Figure S6, Table S2), something that cannot be found from a linear analysis. This skewed output implies that the underarm throw could be perceived as a weaker throw because the projectile's most likely landing location undershoots the target by 4 times as much as the overarm throw.

Table S2. Comparison of linear and nonlinear calculations for bin at $l = 2$, $h = -1$. Dimensional estimates for an arm length of 1m.

Linear	Nonlinear				
$\dfrac{\lambda_{\text{over}}}{\lambda_{\text{under}}}$	$\dfrac{\sigma_{\text{over}}}{\sigma_{\text{under}}}$	Mean landing location		Most likely landing location	
		Overarm	Underarm	Overarm	Underarm
0.69	0.56	1.99	2.01	1.98<br>2 cm undershoot	1.92<br>8 cm undershoot

Linear: $\lambda(\phi^{\text{optim}}_{\text{over}}) = 1.35$, $\lambda(\phi^{\text{optim}}_{\text{under}}) = 1.96$; Nonlinear: $\sigma_{\text{over}} = 0.19$, $\sigma_{\text{under}} = 0.34$.

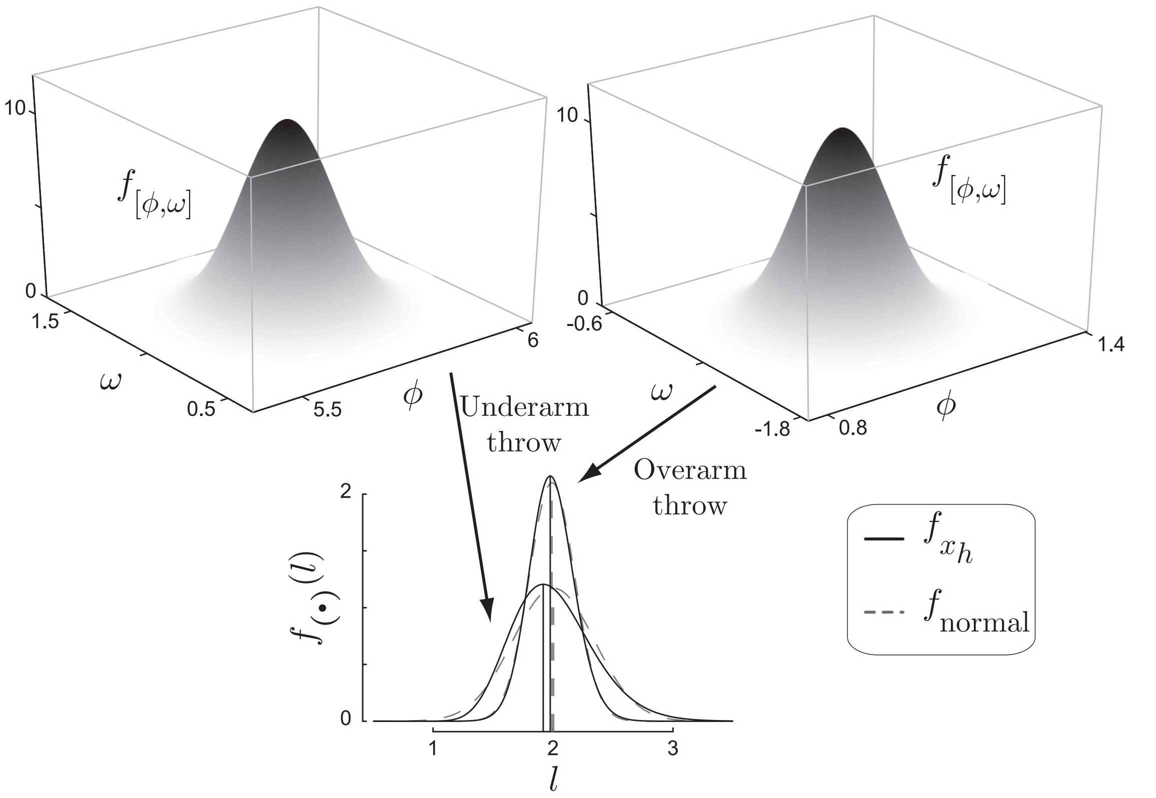

Figure S6. Propagating initial distributions with equal variance for hitting a target at $l = 2$, $h = -1$. The underarm throw amplifies errors more than the overarm throw, in agreement with the linear analysis. For comparison, we superimpose a normal distribution (dashed curves) with mean and variance equal to the propagated distribution (solid curves), together with the mode (solid vertical line) and mean (dashed vertical line). The overarm throw leads to a nearly normal distribution where the projectile lands with mode (1.98) and mean (1.99) nearly equal to each other. The underarm throw in contrast leads to a skewed distribution where the most likely outcome is an undershoot as seen from the mode (1.92) although the mean (2.01) is nearly at the target ($l = 2$). The input distributions are centred at ($\phi_{\text{over}}^{\text{optim}} = 1.082$, $\omega_0(\phi_{\text{over}}^{\text{optim}}) = -1.186$) for the overarm throw and at ($\phi_{\text{under}}^{\text{optim}} = 5.716$, $\omega_0(\phi_{\text{under}}^{\text{optim}}) = 1.008$) for the underarm throw, and we set standard deviations as $\sigma_\phi = 0.087$, $\sigma_\omega = 0.177$ for both throws. For a one metre long arm, these values expressed as mean $\pm$ standard deviation are, overarm: $\phi = 62 \pm 5°$, $|v| = 3.72 \pm 0.56\,\text{m/s}$ ($13.4 \pm 2\,\text{km/hr}$) and underarm: $\phi = -33 \pm 5°$, $|v| = 3.17 \pm 0.56\,\text{m/s}$ ($11.4 \pm 2\,\text{km/hr}$).

S9 Shooting: Zero arm length

Our considerations so far use the length of the arm R to set the length scale in the problem. We now look at the limit when this length scale becomes vanishingly small

or for throwing to faraway targets where $L, H \gg R$. In this, the artillery limit, the problem becomes one of shooting a projectile at an angle θ with a linear velocity V. Naturally therefore, we only consider targets at the same height as the origin from where the projectile is launched. The ratio of variability in V to the variability in θ, introduces a velocity scale $k = \delta v/\delta\theta$, where the quantities δv and $\delta\theta$ are appropriate measures of variability. By expressing distances in units of k^2/g and time in units of k/g, we obtain the scaled trajectory equations for distance x, height y, as a function of time t, launch angle θ and launch velocity v,

$$x(t) = tv\cos\theta \tag{S9.1a}$$

$$y(t) = tv\sin\theta - \frac{1}{2}t^2. \tag{S9.1b}$$

For exactly striking the target at a distance l with the launch parameters θ, v, the projectile lands at $x_0(\theta, v) = l = gL/k$. This gives the one-dimensional curve of launch parameters $(\theta_0, v_0(\theta_0))$:

$$v_0(\theta) = \sqrt{\frac{l}{\sin 2\theta}}. \tag{S9.2}$$

Performing a linearized error analysis in the neighbourhood of this curve $(\theta_0, v_0(\theta_0))$ yields the Jacobian J that maps small errors $\delta\theta$ and δv to small errors δx_0:

$$J(\theta_0) = \begin{pmatrix} \frac{\partial x_0}{\partial\theta} & \frac{\partial x_0}{\partial v} \end{pmatrix}\Bigg|_{\theta=\theta_0, v=v_0(\theta_0)} = \begin{pmatrix} 2v_0^2\cos 2\theta & 2v_0\sin 2\theta \end{pmatrix} = 2\begin{pmatrix} \frac{l\cos 2\theta}{\sin 2\theta} & \sqrt{l\sin 2\theta} \end{pmatrix}. \tag{S9.3a}$$

The shooting angle most robust to small input noise corresponds to the θ where the only positive singular value is minimized. Minimizing the square of the singular value

$$\zeta(\theta) = J^{\mathrm{T}}J = \frac{l^2\cos^2 2\theta}{\sin^2 2\theta} + l\sin 2\theta \tag{S9.4}$$

yields

$$\left.\frac{d\zeta}{d\theta}\right|_{\theta=\theta^*} = 0 \Rightarrow 4l\cos 2\theta^* - 8l^2\frac{\cos 2\theta^*}{\sin^3 2\theta^*} = 0 \tag{S9.5a}$$

$$\Rightarrow \cos 2\theta^* = 0,\ \text{or, for } 0 < l < \frac{1}{2},\ \sin 2\theta^* = \sqrt[3]{2l} \tag{S9.5b}$$

We therefore find that for $l > 1/2$, there is only one extremum at $\theta^* = \pi/4$, but three extrema co-exist for $0 < l < 1/2$ at $\theta^* = \pi/4$, $\arcsin\sqrt[3]{2l}/2$, and $\pi/2 - \arcsin\sqrt[3]{2l}/2$. To

determine if the solution [**S9.5b**] is a minimum or maximum, we evaluate

$$\frac{d^2\zeta}{d\theta^2} = -16l\sin 2\theta + \frac{32l^2 + 64l^2\cos^2 2\theta}{\sin^4 2\theta} \tag{S9.6a}$$

$$\Rightarrow \left.\frac{d^2\zeta}{d\theta^2}\right|_{\theta=\theta^*} = \begin{cases} 16l(2l-1) & \text{when } \theta^* = \frac{\pi}{4},\ 0 < l \\ 24(2l)^{\frac{2}{3}}(1-(2l)^{\frac{2}{3}}) & \text{when } \sin 2\theta^* = \sqrt[3]{2l},\ 0 < l < \frac{1}{2} \end{cases} \tag{S9.6b}$$

From the solutions in [**S9.6b**] we see that $\theta^* = \pi/4$ is a local minimum for $l > 1/2$, a local maximum for $l < 1/2$, and there is a pitchfork bifurcation at $l = 1/2$. We also find that for $l < 1/2$, when $\theta^* = \pi/4$ becomes a local maximum, there are two new branches that are a local minimum, with a cubic dependence on l near $l = 1/2$.

The result that the optimal shooting angle (θ^*) for targets with $l > 1/2$ is $\pi/4$ is well known, including cases where there is air drag [2]. However these past results however do not scale the equations like we do using the relative amount of noise in shooting velocity compared to shooting angle, and therefore do not identify the pitchfork bifurcation. Thus $\theta = \pi/4$ becomes the worst possible choice for shooting when $l < 1/2$, and there exist two symmetric branches of optimal shooting angles.

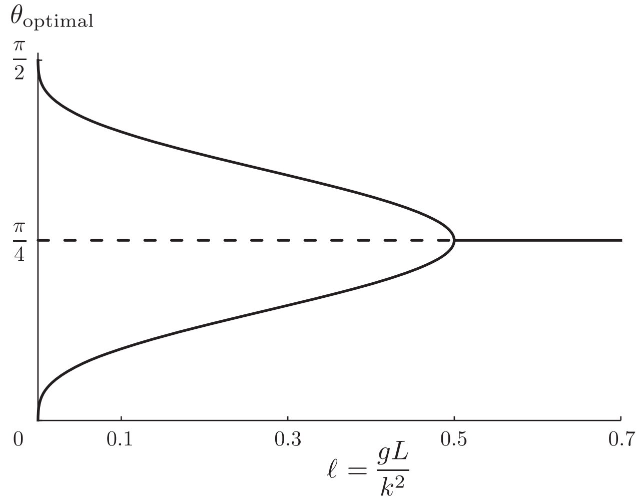

Figure S7. Bifurcation diagram for best shooting strategy based on a linearization in the neighborhood of all strategies that lead to an accurate strike. Recall that $l = gL/k^2$ is the dimensionless distance to the target and θ_{optimal} is the angle to shoot the target so that launching errors are amplified the least amount.

To understand this result, we first consider the limit of the relative noise in shooting velocity being much greater than that in the shooting angle, i.e. $k \gg 1 \Rightarrow l \approx 0$. Then the best shooting angle corresponds to zero sensitivity to fluctuations in velocity, namely a high velocity shot with $\theta = 0$ or $\theta = \pi/2$. At the other extreme where $k \approx 0 \Rightarrow l \gg 1$,

i.e. when the noise in shooting angle is much larger than in shooting velocity, $\pi/4$ is the optimal angle because that corresponds to a local maximum for projectile range, and hence has zero sensitivity to fluctuations in shooting angle. There is a bifurcation, and not a smooth continuous change between these two extreme cases because the problem is symmetric about $\pi/4$ and the optimal shooting angle depends continuously on l. The specific bifurcation point is determined by a trade off between the two sensitivities of the shooting range to shooting angle and velocity, respectively. For small perturbations ϵ near $\theta = \pi/4$, the sensitivity to shooting angle is $16l^2\epsilon^2 + O(\epsilon^3)$ and the sensitivity to shooting velocity is $-8l\epsilon^2 + 4l + O(\epsilon^3)$. Therefore, in the neighbourhood of $\theta = \pi/4$ and for $l \leq 1/2$, the sensitivity to shooting velocity overpowers the sensitivity to shooting angle.

Supplementary references

1. W. T. Dempster. Space requirements of the seated operator: geometrical, kinematic, and mechanical aspects of the body, with special reference to the limbs. Technical Report Contract No. AS 18(600)-43 Project No. 7214, University of Michigan, Aero Medical Laboratory Wright Air Development Center Air Research and Development Command United States Air Force Wright-Patterson Air Force Base, Ohio, July 1955. URL `http://hdl.handle.net/2027.42/4540`.

2. J. M. Gablonsky and A. Lang. Modeling Basketball Free Throws. *SIAM Review*, 47 (4):775–798, 2005.

3. K. Mardia and P. Jupp. *Directional statistics*. Wiley New York, 2000.

4. C. L. Ogden, C. D. Fryar, M. D. Carroll, and K. M. Flegal. Mean body weight, height, and body mass index, united states 1960-2002. *Adv Data*, (347):1–17, Oct 2004.

5. H. Okubo and M. Hubbard. Dynamics of the basketball shot with application to the free throw. *J Sports Sci*, 24(12):1303–1314, 2006.

6. A. Tan and G. Miller. Kinematics of the free throw in basketball. *American Journal of Physics*, 49(6):542–544, 1981.

7. R. von Mises. Über die "Ganzzahligkeit" der Atomgewichte und verwandte Fragen. *PhysikalischZe.*, 19:490–500, 1918.

8. G. C. Westergaard, C. Liv, M. K. Haynie, and S. J. Suomi. A comparative study of aimed throwing by monkeys and humans. *Neuropsychologia*, 38(11):1511–1517, 2000.
